# The Maize Sulfite Reductase Is Involved in Cold and Oxidative Stress Responses

**DOI:** 10.3389/fpls.2018.01680

**Published:** 2018-11-15

**Authors:** Zongliang Xia, Meiping Wang, Ziwei Xu

**Affiliations:** ^1^College of Life Science, Henan Agricultural University, Zhengzhou, China; ^2^Synergetic Innovation Center of Henan Grain Crops and State Key Laboratory of Wheat and Maize Crop Science, Zhengzhou, China; ^3^Department of Information, Library of Henan Agricultural University, Zhengzhou, China

**Keywords:** maize, sulfite reductase, cold stress, oxidative stress, glutathione

## Abstract

Sulfite reductase (SiR) functions in sulfate assimilation pathway. However, whether it is involved in stress response in crops is largely unknown. Here, the *SiR* ortholog from *Zea mays* (*ZmSiR*) was characterized. The recombinant ZmSiR protein was purified from *E. coli*. It exhibited sulfite-dependent activity and had strong affinity for sulfite. *ZmSiR* transcripts were markedly up-regulated by cold and methyl viologen (MV) treatments. Overexpression of *ZmSiR* complemented growth retardation phenotype of *Arabidopsis atsir* mutant. *ZmSiR*-overexpressing *Arabidopsis* plants were tolerant to severe SO_2_ stress and rescued the susceptible phenotype of the *atsir. ZmSiR* knock-down transgenic maize plants with 60% residual transcripts were more susceptible to cold or oxidative stress than wild-type. The severe damage phenotypes of the *ZmSiR*-compromised maize plants were accompanied by increases of sulfite and H_2_O_2_ accumulations, but less amounts of GSH. The qPCR analysis revealed that there was significantly altered expression of several key sulfur metabolism-related genes in *ZmSiR*-impaired maize lines under cold or MV stress. Particularly, *ZmAPR2* expression was significantly elevated, suggesting that toxic sulfite accumulation in *ZmSiR*-impaired plants could be attributable to the reduced *SiR* coupled to increased *ZmAPR2* expression. Together, our results indicate that *ZmSiR* is involved in cold and oxidative stress tolerance possibly by modulating sulfite reduction, GSH-dependent H_2_O_2_ scavenging, and sulfur-metabolism related gene expression. *ZmSiR* could be exploited for engineering environmental stress-tolerant varieties in molecular breeding of maize.

## Introduction

In higher plants, the assimilatory sulfate reduction is an essential metabolic process in which a great number of sulfur-containing amino acids, sulfolipids and coenzymes are synthesized (Leustek and Saito, 1999; Leustek et al., 2000; Lewandowska and Sirko, 2008). This process involves several successive enzymatic reactions that occur in chloroplasts. Firstly, sulfate is transported to chloroplasts, where it is adenylated by ATP sulfate adenylyltransferase (APS) (Cao et al., 2013). Then, the product adenosine-phosphosulfate is reduced to sulfite by APS reductases (APRs). And then, reduction of the toxic sulfite to sulfide is catalyzed by sulfite reductase (SiR). Finally, the sulfide is further incorporated into cysteine by *O*-acetyl-serlyases that can be used for synthesizing other sulfur-containing compounds such as glutathione (Nakayama et al., 2000).

Plant SiR is a kind of soluble protein that contains one iron-sulfur cluster (4Fe-4S) and one siroheme group. It is localized to plastids and catalyzes reduction of sulfite to sulfide using reduced ferredoxin (Fd) as a physiological electron donor (Nakayama et al., 2000). It was evidenced that SiR protected *Arabidopsis* or tomato plants against sulfite/SO_2_ toxicity via sulfite reduction-dependent pathway (Yarmolinsky et al., 2013). Thus, SiR has been thought to be a bottleneck for assimilatory sulfate reduction in plants (Khan et al., 2010). In addition to reductive assimilation, plant SiR plays an important role in normal growth and development. For example, *Nicotiana benthamiana* SiR (NbSiR) protein has been evidenced to act as a chloroplast (cp) nucleoid protein with cp DNA-compacting and transcription-regulatory activities (Sekine et al., 2007; Kang et al., 2010). Furthermore, the conserved C-terminally encoded peptides of the SiR are important to localize to the cp nucleoids in plants (Kobayashi et al., 2016). NbSiR silencing differentially affected expression of plastid-encoded genes and ultimately resulted in chloroplast ablation (Kang et al., 2010), indicating that plant SiR functions in cp-nucleoid metabolism, plastid gene expression, and chloroplast development. In addition, it was reported that *Arabidopsis* plants with impaired SiR expression exhibited dramatically reduced biomass and marked metabolism disturbances (Khan et al., 2010). Also, tomato plants with lowered SiR activity showed clearly reduced biomass and promoted early leaf senescence (Yarmolinsky et al., 2014). These results indicate that SiR is essential for plant growth and development.

Although new insights in biological functions of SiR were reported in model plants, the knowledge of SiR function and regulation from crops is very limited. Environmental stresses such as drought, cold, and high salinity cause oxidative stress by provoking cellular redox imbalances, thus seriously affecting growth, development, and biomass of crop plants (Gechev et al., 2006). To date, information about biochemical properties and biological function of maize SiR is scarce. In this study, we characterize enzymatic properties of the maize SiR and its function in cold and oxidative stresses in maize.

## Materials and Methods

### Plant Material and Growth Conditions

The maize inbred line Zheng58-1 and *Arabidopsis thaliana* ecotype Columbia (Col-0) were used for this study. For *Arabidopsis*, the seeds were surface sterilized and then germinated on plates containing one-half-strength Murashige and Skoog (MS) medium. After 7 days, the seedlings were transferred to sterilized low-nutrient soil to obtain fully grown plants. For maize, the seeds were germinated on moist filter papers in the Petri dishes (120 mm Φ) and then transferred to pots with nutrient soil for culture. Both *Arabidopsis* and maize plants were grown in a growth room at approximately 23°C, 60–70% relative humidity, a photoperiod of 18 h/6 h (day/night) and light intensity of 200 μmol m^-2^ s^-1^, as described previously (Xia et al., 2013).

### Stress Treatments on Maize Plants

For oxidative stress, 2-week-old Zheng 58-1 plants in soil were sprayed with 20 μM of MV. For cold stress, the same age maize plants in soil were transferred to grown at 4°C in the growth chamber. During 48 h of both types 58st NL C-Terminal Region types of stress treatments, leaf samples were harvested at indicated time points (0, 1, 3, 6, 12, 24, and 48 h) for gene expression analyses.

### Overexpression, Purification, Immunoblot Detection, and Kinetic Analysis of Recombinant ZmSiR

The *ZmSiR* full-length cDNA fragment was amplified and cloned into the vector pET-30a (Novagen, United States) with *Nde*I/*Xho*I sites (Supplementary Table S1). The resulting expression construct pET-ZmSiR was transformed into *E. coli* BL21 (DE3) pLysS cells, which could produce a recombinant protein with a 6× histidine tag at its N-terminus by 0.5 mM of isopropylthio-β-galactoside (IPTG) induction. The cells were harvested by centrifugation at 8,000 × *g* for 10 min and resuspended in a solution containing 0.1 M Tris acetate (pH 7.6), 0.5 M sucrose, 0.1 mM DTT, 0.5 mM EDTA, and Protease inhibitor cocktail, and then sonication was conducted using an ultra-sonicator (450 Digital Sonifier, United States) on ice with 50% pulse amplitude. After that, the bacterial fractions containing ZmSiR-His were centrifuged (8,000 × *g*, 20 min) and the precipitations were solubilized at 4°C overnight in a dissolution buffer [0.1 M potassium phosphate buffer (pH 7.4), 10 mM β-mercaptoethanol, 0.5 M NaCl, 20% (w/v) glycerol, l, 1.0% (w/v) CHAPS, and 0.5% (v/v) NP-40]. The purification of ZmSiR-His protein was carried out using a Ni^2+^-affinity purification kit (Sangon, Co., Shanghai, China) as previously described (Xia et al., 2012). Briefly, the solubilized fraction was loaded onto a pre-equilibrated Ni^2+^-affinity column and bound proteins were washed with a washing buffer containing 0.1 M potassium phosphate buffer (pH 7.4), 10 mM β-mercaptoethanol, 0.5 M NaCl, 20% (w/v) glycerol, and 30 mM imidazole. The purified protein was obtained using an elution buffer that had a same composition as the washing buffer, except that 30 mM imidazole was replaced by 20 mM imidazole. The eluted fraction containing the ZmSiR-His protein was collected and dialyzed at 4°C against 100 mM potassium phosphate buffer (pH 7.4) containing 20% (v/v) glycerol.

For immunoblot detection of the recombinant SiR, the cell extracts or purified proteins were separated using 12% SDS-Tris-glycine polyacrylamide gel electrophoresis (SDS-PAGE) and transferred onto polyvinylidene difluoride (PVDF) membranes. The membranes were blocked and thereafter blotted with a commercial His-tagged monoclonal antibody (CWBIO, Co., Beijing, China) for 3 h at a 1:3000 dilution. After extensive washing, the bound primary antibody was detected with a horseradish peroxidase (HRP)-conjugated goat anti-mouse IgG secondary antibody (CWBIO Co., Beijing, China). The HRP activity was visualized using a 3,3′-diaminobenzidine (DAB) development kit (CWBIO, Co., Beijing, China) as described by us (Xia et al., 2012).

Kinetic analysis of ZmSiR was conducted using a saturating concentration of NADPH (400 μM) and varying sulfite concentrations (from 2.5 to 400 μM). The kinetic parameters (*K*_m_ and *V*_max_) were calculated as described previously (Xia et al., 2012). For the assays, three replicates were conducted for each test sample and the experiment was repeated three times.

### Verification of *SiR Arabidopsis* Mutants

The *AtSiR* T-DNA insertion mutant (SALK_047219) (named *atsir-s047*) seeds were obtained from the ABRC collection center (The Ohio State University, Columbus, OH, United States). Homozygous mutants were identified by PCR from genomic DNA using forward primer P1, T-DNA left border primer LBb1, and *AtSiR* (Accession No. At5g04590) gene-specific reverse primer P2 (Supplementary Table S1), and analyzed further by DNA sequencing to confirm the insertions of the T-DNA in the gene. The transcript levels of *AtSiR* in the Col-0 and mutants were determined by quantitative PCR.

### Construction of atsir/35S-ZmSiR Transgenic *Arabidopsis* Plants and SO_2_ Stress Phenotype Analysis

The full-length *ZmSiR* cDNA was amplified by PCR using corresponding primer pairs (Supplementary Table S1), and was subsequently into the binary vector pART27 (Xia et al., 2012) to produce the binary construct pART27-35S-ZmSiR. The construct was then transformed into *atsir-s047 Arabidopsis* plants through the *Agrobacterium*-mediated floral dip method (Clough and Bent, 1998). The positive transgenic lines were confirmed and maintained as described by us (Xia et al., 2012). Homozygous T3 *Arabidopsis* lines were used for phenotypic analysis.

Five-weeks old wild-type, *atsir* mutant, and atsir/35S-ZmSiR transgenic plants were exposed to 6 ppm SO_2_, or to filtered pollutant-free air (control) for 2 h in fumigation chambers. The gas was released through a tube from a cylinder and kept constant by a SO_2_ monitor in the chamber as described by us (Xia et al., 2015). After treatments, the plants were transferred to the growth room for recovery. Three replicates each consisting of three pots of seedlings from each line were included for both SO_2_ treatment and non-treated controls. The whole experiment was repeated three times.

### Generation and Molecular Verification of *ZmSiR-Compromised* Transgenic Maize Lines

For the *ZmSO-compromised* construct, a 531-bp-length PCR product of *ZmSiR* cDNA was amplified using primers ZmSiR-F2 and ZmSiR-R2 (Supplementary Table S1) and introduced as sense and anti-sense into the binary vector pFGC491 with *Bam*HI and *Xba*I, or NcoI and SwaI restriction sites, resulting in the transformation construct pFGC491-35S: ZmSiR-RNAi. The recombinant plasmid was introduced into the *A. tumefaciens* strain *LBA4404*, which was used to transform maize. Transformation of maize inbred line Zheng58-1 immature embryos was conducted as described by us previously (Xia et al., 2015). Transgenic maize seedlings with Basta resistance were screened and confirmed by PCR as described previously (Xia et al., 2015). The number of T-DNA inserts in PCR-positive plantlets was assessed by segregation of T_0_ offspring on selective medium. Several T_0_ seedlings of each one-copy line were grown in the field and their seeds were germinated and used for stress assays. The transcript levels of *ZmSiR-compromised* transgenic lines were evaluated by RT-qPCR as described below.

### Analysis of *ZmSiR-Compromised* Maize Lines for Oxidative or Cold Stress Tolerance

For oxidative stress, 3-week-old maize WT and *ZmSiR-compromised* lines (S2, S5, and S6) were sprayed with 20 μM of MV. Three replicates each consisting of two pots of seedlings from each line were included for both MV treatment and H_2_O-treated controls. For cold stress, 12-day-old WT and both *ZmSiR-compromised* lines (S5 and S6) were transferred to grow hydroponically in MS liquid medium at 4°C (Cold) or 23°C (Control) for 3 days. After treatments, phenotypes of various types of plants were photographed. Total chlorophyll content in both WT and RNAi plants was determined as described by Arnon (1949). In brief, leaves were sampled and ground in 80% acetone and the homogenate was centrifuged. Then, absorbance of the supernatant was measured at 645 and 663 nm using a spectrophotometer (Hitachi U2000, Japan). Finally, the relative remaining chlorophyll (%) of the WT and both RNAi maize lines was determined. The experiments were repeated three times each.

### Histochemical and Quantified Detection of H_2_O_2_ Accumulation

The histochemical detection of H_2_O_2_ from MV-treated maize seedlings with 3, 3′- diaminobenzidine (DAB) was performed according to our previous method (Xia et al., 2013). After staining, the leaf disks were rinsed in 80% (v/v) ethanol for 10 min at 70°C, mounted in lactic acid:phenol:water (1:1:1, v/v/v), and photographed directly using a digital camera. H_2_O_2_ content in various genotypes of maize plants was assayed according to our published method (Xia et al., 2012).

### Determination of SiR Activity, Sulfite, and Glutathione Contents

Sulfite reductase activity assay was determined as described by Khan et al. (2010). Sulfite and reduced glutathione (GSH) contents of leaves were measured using ion-exchange chromatograph system as described by us (Wang et al., 2016). For each assay, three replicates were done for each test sample and these experiments were repeated three times.

### Quantitative Real-Time PCR (RT-qPCR)

Total RNA extraction was conducted as described previously (Xia et al., 2012). The first-strand cDNA was synthesized from 1 μg of total RNA using the SuperScript^TM^ RT kit (Thermo Scientific, United States) in a 10 μL-reaction volume according to the manufacturer’s recommendations. qPCR was performed in 96-well white plates using an IQ5 Real Time PCR (Bio-Rad, Hercules, CA, United States) with three biological replicates and three technical replicates. The 20 μL reaction mixture consisted of 1 μL cDNA diluted 10-fold, 10 μL master mix (SYBR Green Supermix, Thermo Scientific, United States), and 0.5 μM of each gene-specific primer (Supplementary Table S1). The qPCR procedures were performed according to our detailed descriptions (Su et al., 2017). Data were normalized to the corresponding reference genes transcripts (*AtActin2* and *AtTubulin* for *Arabidopsis*, and *ZmActin2* and *ZmTubulin* for maize) and the transcript levels of genes were calculated according to the 2^-ΔΔCt^ method (Livaka and Schmittgen, 2001). All qPCR experiments followed the MIQE guidelines (Bustin et al., 2009).

### Statistical Analysis

The data in this study are presented as the mean ± SE of three replicates and were analyzed by a simple variance analysis (ANOVA). The differences between the means were compared by Student’s *t*-test at *P* < 0.05. SPSS statistical software (version 17.0) was used to perform the analyses.

### Accession Numbers

Sequence data in this study can be found in the GenBank under the following Accession Nos. *ZmSO*, FJ436404*; ZmSiR*, NP_001105302; *ZmAPR2*, GRMZM2G087254; *ZmCys2*, LOC109939155; *ZmSQD1*, LOC100282424; *ZmSULTR1;1*, GRMZM2G 159632; *ZmSULTR3;1*, GRMZM2G154211; *ZmGSH1*, LOC103648180; *ZmGSH2*, LOC542055.

## Results

### Molecular Characterization of Maize Sulfite Reductase Gene (*ZmSiR*)

**FIGURE 1:**
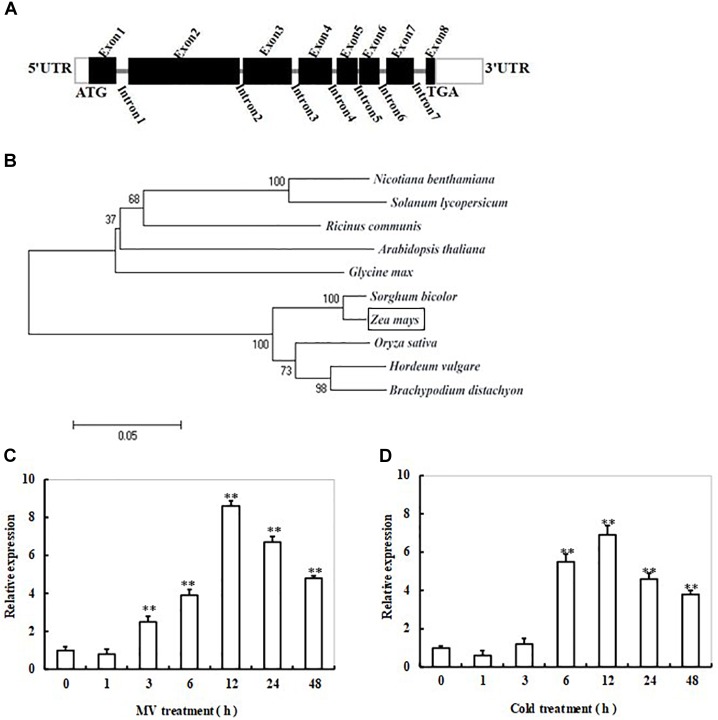
Gene structure, phylogenetic tree, and transcript profiles of *ZmSiR* in response to cold or oxidative stress in maize. **(A)** Schematic representation of the ZmSiR gene structure. The thick black boxes represent exons with the predicted translation start site (ATG) and stop codon (TGA); the gray bars represent introns. White boxes indicate the 5′- and 3′-untranslated regions. **(B)** Phylogenetic tree based on the amino acid sequence alignment of plant SiR. These plant species include *Arabidopsis thaliana* (CAA89154.1), *Solanum lycopersicum* (AFB83709.1), *Ricinus communis* (XP_002513495.1), *Nicotiana benthamiana* (ACN23794.1), *Glycine max* (XP_003540209.1), *Sorghum bicolor* (XP_002441346.1), *Oryza sativa* (NP_001055978.1), *Hordeum vulgare* (BAK03240.1), *Brachypodium distachyon* (XP_003568157.1), and *Zea mays* (NP_001105302.1). The bootstrap values shown were calculated based on 500 replications. The tree was constructed using the neighbor-joining method. **(C)** Time-course analysis of *ZmSiR* transcript levels in maize seedlings under MV-induced oxidative stress. **(D)** Time-course analysis of *ZmSiR* transcript levels in maize seedlings under cold stress. In both **(C,D)** assays, 2-week-old maize seedlings were exposed to 20 μM methyl viologen (MV) or 4°C for indicated time points (0, 1, 3, 6, 12, 24, and 48 h), and leaf samples were harvested for RT-qPCR analysis. Relative expression levels of *ZmSiR* were normalized to the internal control gene *ZmActin2* and compared with the value for samples prior to treatments (Time 0 h). Data are the mean ± SE from three independent experiments. ^∗∗^*t*-test, with *P* < 0.01.

At the start of this work, total RNAs from 2-week-old maize plants, which had been treated with 20 μM methyl viologen (MV) for 12 h, were used as samples for transcriptome analysis to identify genes responsive to oxidative stress. A gene encoding sulfite reductase in maize (named *ZmSiR*) with nearly 10-fold induction was observed (data not shown). The full-length cDNA of *ZmSiR* from maize was obtained by RT-PCR. The cDNA sequence of *ZmSiR* is 2370 bp long, which consists of a 94 bp 5′-untranslated region, a 368 bp 3′-untranslated region, and a 1908 bp ORF. Sequence comparison between gDNA and cDNA revealed that the entire *ZmSiR* genomic sequence contains seven introns and eight exons that encode a 635 amino acid polypeptide (Figure 1A). Amino acid sequence comparisons have revealed that ZmSiR exhibits high identity to counterpart proteins from *Arabidopsis thaliana* (71% identity), *Solanum lycopersicum* (65% identity), *Ricinus communis* (67% identity), *Nicotiana benthamiana* (66% identity), *Glycine max* (68% identity), *Oryza sativa* (91% identity), *Brachypodium distachyon* (89% identity), *Hordeum vulgare* (89% identity), and *Sorghum bicolor* (97% identity) (Supplementary Table S2).

A phylogenetic tree was established based on SiR protein sequences available in GenBank from 10 plant species including *Arabidopsis*, tobacco, tomato, soybean, castor, rice, barley, wild wheat, sorghum, and maize (Figure 1B). As shown in Figure 1B, these SiRs were clustered into two distinct groups, monocot and dicot SiR groups. In the monocot SiRs, the SiR from maize showed higher identities with sorghum, and thus was clustered into the same isoform subgroup. On the other hand, the SiRs from rice, barley and wild wheat formed another subgroup. In the dicot SiRs, the SiRs from tobacco and tomato were clustered into the same isoform subgroup, while the SiRs from other three species *Arabidopsis*, soybean and castor formed another subgroup (Figure 1B). These results clearly demonstrated that ZmSiR is highly similar to the known SiR proteins from *Arabidopsis* and other plant species, thus could be identified as an ortholog of SiR.

### Transcript Profiles of *ZmSiR* in Response to Oxidative or Cold Stress

Time-course analysis of *ZmSiR* transcript levels in maize plants in response to oxidative stress was performed by qPCR. As shown in Figure 1C, the transcript level of *ZmSiR* was increased rapidly at 3 h, and reached a maximal level at 12 h (about eightfold increase in transcripts) during 48 h of MV treatment (Figure 1C). This suggests that *ZmSiR* could be involved in oxidative stress response.

Also, transcriptional response of *ZmSiR* to cold stress in maize plants was examined by qPCR. The expression level of *ZmSiR* displayed a significant increase at 6 h of cold stress, and reached a maximal level at 12 h (about sixfold increase in transcripts), and then gradually decreased, finally maintained to a relatively high level during 48 h of the treatment (Figure 1D). This result indicates that *ZmSiR* is also responsive to cold stress.

### Sulfite Reductase Activity of ZmSiR *in vitro*

**FIGURE 2:**
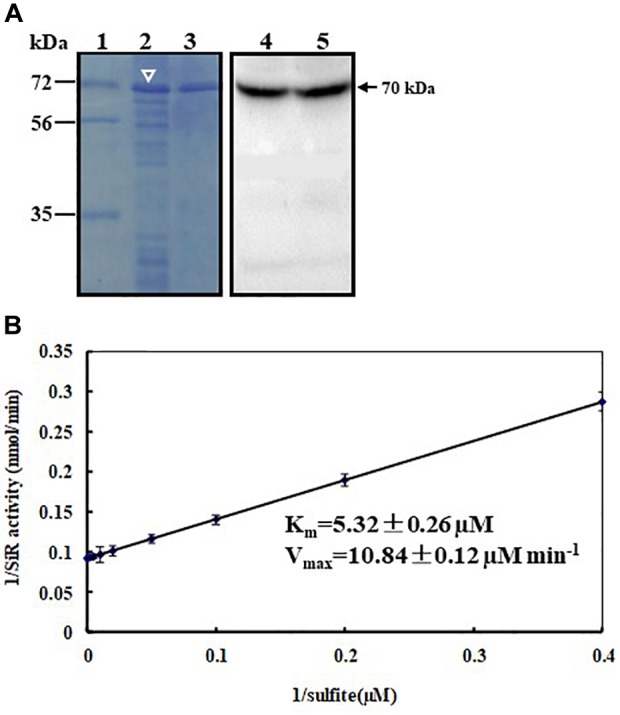
Expression, purification, and kinetic analysis of recombinant maize sulfite reductase. **(A)** Purification of histidine-tagged maize SiR. The histidine-tagged ZmSiR (with a predicted molecular mass of 70 kDa) was expressed in bacterial cells induced by 0.5 mM isopropyl-β-D-thiogalactopyranosid (IPTG) (lane 2, indicated by white arrowhead). The overexpressed protein was purified using Ni^2+^-affinity chromatography (lane 3). The overexpressed and purified SiR proteins were blotted with an anti-histidine monoclonal antibody and the blotted target bands were visualized using a 3,3′-diaminobenzidine (DAB) development kit based on the HRP activity (lane 4 and lane 5; signals marked by an arrowhead). The molecular mass markers (kDa, lane 1) are shown on the left. **(B)** Steady-state kinetics of recombinant ZmSiR with sulfite. Double-reciprocal presentation (Lineweaver–Burk plot) of enzyme rate was conducted using varying concentrations of sulfite (2.5, 5, 10, 25, 50, 100, 200, and 400 μM) and constant 400 μM NADPH. The reaction was initiated with 1.0 μg of purified recombinant ZmSiR.

To ascertain whether the cloned *ZmSiR* encodes a functional sulfite reductase, we have induciblly over-expressed the hexahistidine (His)-tagged ZmSiR protein in *E. coli* and purified it using Ni^2+^-affinity chromatography (Figure 2A; left panel). The purified ZmSiR protein had a MW of 70 kDa as shown by Western blot assays. This mass was in agreement with our prediction (Figure 2A; right panel). Subsequently, biochemical assays were conducted to analyze its kinetics, in which the recombinant ZmSiR protein showed a sulfite-dependent activity when NADPH acted as an electron acceptor. In the assay, the Michaelis constant *K*_m_ for sulfite was calculated to be 5.32 ± 0.26 μM, and the maximum velocity *V*_max_ for sulfite was calculated to be 10.84 ± 0.12 μM min^-1^ (Figure 2B). Similar kinetic parameters were also produced in our independent biological replicates. These results suggested that ZmSiR has sulfite reductase activity *in vitro*.

### *ZmSiR* Could Complement Growth Retardation Phenotypes of *Arabidopsis SiR* Mutant

To clarify whether *ZmSiR* gene is a functional ortholog of *Arabidopsis SiR*, one T-DNA insertion line SALK_047219 (named *atsir-s047*) was identified in the ABRC seed stock center. In this line, the T-DNA was inserted in the promoter region of *AtSiR* (Supplementary Figure S1A). Noticeably, the T-DNA lines located in the gene coding region were embryo lethal (Khan et al., 2010). The T-DNA insertion position is illustrated in Supplementary Figure S1A, and homozygous and heterozygous mutants were verified using *AtSiR* gene-specific and T-DNA border primers. In accordance with previous findings by Khan et al. (2010), the *atsir-s047* homozygous line also showed retarded growth phenotypes (Supplementary Figure S1E). qPCR assays revealed that a decrease of 50% approximately in mRNA abundance was detected compared with wild-type plants of the same age (Supplementary Figure S1B). Accordingly, the SiR activity also was lowered to about 50% of the wild-type level (Supplementary Figure S1C).

To verify whether *ZmSiR* could complement growth retardation phenotypes of the *atsir-s047* mutant, the construct harboring the complete ORF of *ZmSiR* was transformed into *atsir-s047* mutant, and two homozygous transgenic *Arabidopsis* lines (atsir/35S- ZmSiR# 3 and 7) were used for further analysis. The enzymatic activity determination showed that these transgenic lines had higher SiR activities than the WT, except for the mutant *atsir-s047* (Supplementary Figure S1D). Under normal conditions, the *atsir-s047* plants showed clearly retarded growth phenotypes compared to the WT, whereas both transgenic lines (atsir/ 35S-ZmSiR#3 and 7) grew normally, similar to the WT (Supplementary Figure S1E). Further biomass determination showed that both complemented *Arabidopsis* lines had increased by 30–40% in dry weight per plant, compared with the WT (Supplementary Figure S1F). These results suggest that ZmSiR could effectively complement growth retardation phenotypes of the *atsir* mutant *Arabidopsis*, which was due to deficiency of SiR activity.

### *ZmSiR* Could Rescue Phenotypes of *Arabidopsis SiR* Mutant Plants Susceptible to Toxic SO_2_ Stress

**FIGURE 3:**
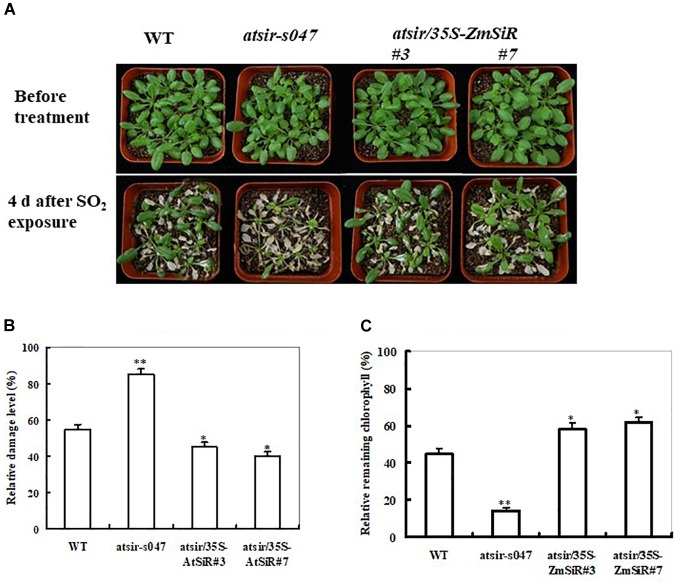
Overexpression of *ZmSiR* in the *atsir* mutant *Arabidopsis* and its response to toxic levels of SO_2_ exposure. **(A)** Toxic effect of SO_2_ on *atsir-s047* and transgenic *Arabidopsis* plants. Five-week-old *WT*, *atsir-s047* and both *atsir/35S-ZmSiR* lines (#3 and #7) were exposed to 6 ppm of SO_2_ for 2 h and examined 4 days later. **(B)** Relative damage levels in the *atsir-s047* and both transgenic *Arabidopsis* lines after SO_2_ exposure. **(C)** Relative residual chlorophyll content in the wild-type, *atsir-s047* and *atsir/35S-ZmSiR Arabidopsis* plants after SO_2_ exposure. Data are means of three independent repetitive experiments ( ± SE), *n* = 20. *^∗∗^t*-test, with *P* < 0.01 and ^∗^*t*-test, with *P* < 0.05.

A previous study has evidenced that *SiR-*impaired *Arabidopsis* plants are susceptible to excess SO_2_ because of loss of sulfite reduction-dependent sulfite detoxifying capability (Yarmolinsky et al., 2013). In view of this, it was interesting to examine whether ZmSiR could rescue phenotypes of *Arabidopsis SiR* mutant susceptible to toxic SO_2_. The *WT*, *atsir-s047*, and both *atsir/35S-ZmSiR* lines (#3 and #7) were exposed to 6 ppm of SO_2_ for 2 h. After 4 days recovery, three types of *Arabidopsis* plants all displayed severe leaf injuries (Figure 3A). Comparatively, both *ZmSiR*-transgenic *Arabidopsis* lines showed relatively less necrosis and chlorophyll bleaching than the *atsir-s047* and wild-type plants (Figure 3A). The leaf damage levels of both *atsir/35S-ZmSiR* lines were much lower than that in the *atsir-s047* line (Figure 3B). Also, the index of residual chlorophyll among these types of plants showed similar changes (Figure 3C). These results have clearly demonstrated that *ZmSiR*-overexpressing transgenic plants, as opposed to *atsir* mutant plants, were tolerant to severe SO_2_ stress and could effectively rescue the susceptible phenotypes of the *atsir* mutant *Arabidopsis*.

### Generation of *ZmSiR-Compromised* Transgenic Maize Lines and Their Response to Oxidative Stress

**FIGURE 4:**
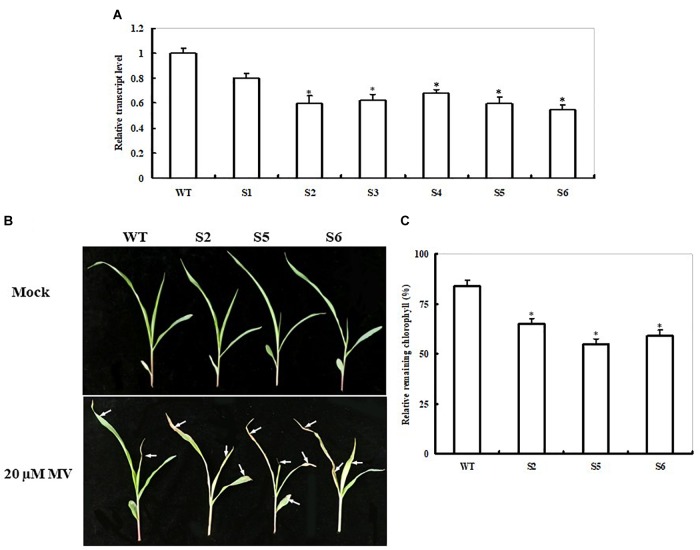
Responses of non-transgenic and *ZmSiR-compromised* transgenic maize lines to oxidative stress. **(A)** Transcript levels of *ZmSiR* in non-transgenic control (WT) and *ZmSiR-compromised* maize lines. Six transgenic lines (S1–S6), along with the WT, were detected by qPCR. **(B)** Toxic effect of MV on WT and *ZmSiR-compromised* maize plants. Three-week-old WT and three *ZmSiR-compromised* lines (S2, S5, and S6) were sprayed with 20 μM of MV and examined 4 days later. **(C)** Relative residual chlorophyll (%) in the wild-type and *ZmSiR-compromised* lines after MV treatment. Values are mean ± SE, *n* = 15. ^∗^*t*-test, with *P* < 0.05.

To further explore *ZmSiR* functions, we employed the transgenic approach to silence the gene in maize. A total of 10 independent maize transgenic lines were generated, and the transgenes were confirmed by PCR (data not shown), six of which (S1–S6) were characterized in more detail. As shown in Figure 4A, the decreases in the *ZmSiR* transcript levels were observed among the six lines by qPCR. Compared with the non-transgenic control (WT), the transcript levels among the six *ZmSiR* RNAi lines decreased by 20–45% (Figure 4A). Among the six lines, three lines (S2, S5, and S6) with sufficient seeds were chosen for further experiments.

Seeds of the three RNAi maize lines were directly sown in soil for the MV treatment experiment. Under normal conditions, these three RNAi lines showed slight growth retardation at the four-leaf seedlings stage compared to WT plants (Figure 4B). Four days after MV treatment, the RNAi transgenic lines showed relative higher necrosis and wilting than the WT plants (Figure 4B). Accordingly, remaining chlorophyll content in these three RNAi lines was significantly lower (35, 45, and 41% decreases, respectively) than that in the WT plants (16% decrease) (Figure 4C). This result showed that impairment of *ZmSiR* in maize plants also decreased tolerance to MV-induced oxidative stress.

### Accumulations of Reactive Oxygen Species and S-Metabolites in *ZmSiR-Compromised* Maize Lines Under Oxidative Stress

**FIGURE 5:**
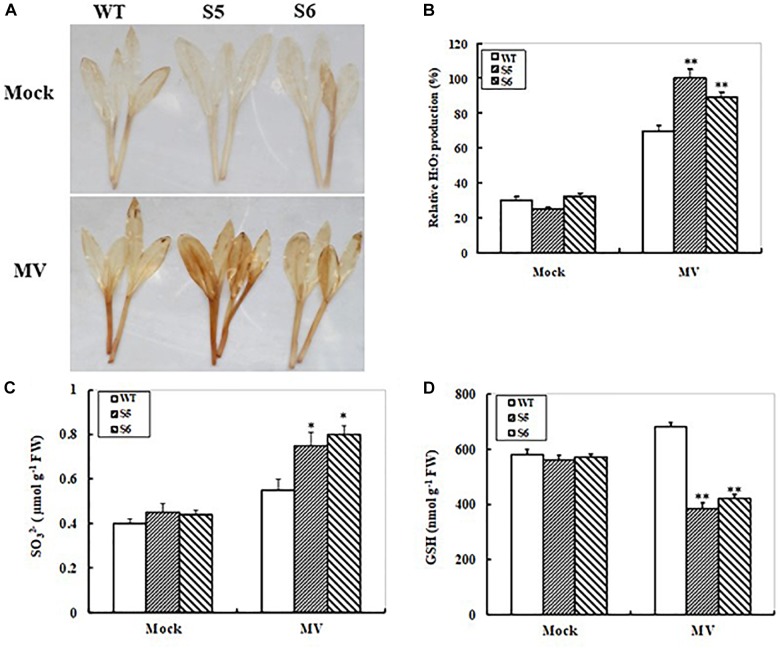
Hydrogen peroxide, sulfite, and glutathione accumulations in non-transgenic and *ZmSiR-compromised* transgenic maize lines upon MV exposure. **(A)** Hydrogen peroxide accumulation detected by *in situ* DAB staining. Ten days old maize seedlings from wild-type and *ZmSiR-compromised* lines (S5 and S6) were treated with distilled water (Mock) or 20 μM MV for 24 h, and were stained by DAB staining. **(B)** Relative H_2_O_2_ levels were quantified in the wild-type and both *ZmSiR-compromised* lines exposed to 20 μM MV for 24 h. Bars indicate SE (*n* = 10). ^∗∗^*t*-test, with *P* < 0.01. **(C)** Sulfite content was determined 24 h after MV (20 μM) spraying. Each experiment was repeated three times. Bar indicates SE. Values are mean ± SE, *n* = 10. ^∗^*t*-test, with *P* < 0.05. **(D)** Glutathione content was measured 24 h after MV (20 μM) spraying. Each experiment was repeated three times. Bar indicates SE. Values are mean ± SE, *n* = 10. ^∗^*t*-test, with *P* < 0.05.

We further detected reactive oxygen species (ROS) accumulations in the WT and RNAi lines during oxidative stress. Histochemical detection of H_2_O_2_ in MV-treated and control maize seedlings from 10-day-old WT and both RNAi lines was performed with DAB staining. No significant differences in DAB staining intensity were observed in WT and both RNAi lines after 24 h of distilled water treatment. When the seedlings were treated with MV, significant increases in DAB staining intensity were observed in both WT and RNAi lines (Figure 5A). However, both RNAi lines showed much higher DAB staining intensity than the WT plants (Figure 5A). Furthermore, quantitative determination of H_2_O_2_ accumulation showed that both RNAi maize lines accumulated higher levels of H_2_O_2_ (2- and 1.8-fold increases, respectively) relative to WT (1.3-fold increase only) after MV treatment (Figure 5B).

To monitor changes in substrate and product levels in maize under oxidative stress, sulfite and reduced glutathione (GSH) contents were determined in MV-treated and control maize seedlings from WT and both RNAi lines. Upon MV exposure, both RNAi lines showed significant increases (67 and 82% increases for S5 and S6, respectively) in leaf sulfite levels, whereas WT plants only increased by 38% (Figure 5C). The reduced glutathione (GSH) levels under oxidative stress were also measured. As shown in Figure 5D, both RNAi maize lines exhibited relatively bigger decreases (31 and 27% decreases for S5 and S6, respectively) (Figure 5D). By contrast, the GSH levels in the WT plants increased by 17% upon MV exposure (Figure 5D). These results indicated that reduced *ZmSiR* expression in maize plants resulted in insufficient GSH levels, which caused more ROS accumulations and oxidative damage upon MV exposure.

### Changes in Sulfur-Metabolism Related Gene Transcripts in *ZmSiR-Compromised* Maize Lines Under Oxidative Stress

**FIGURE 6:**
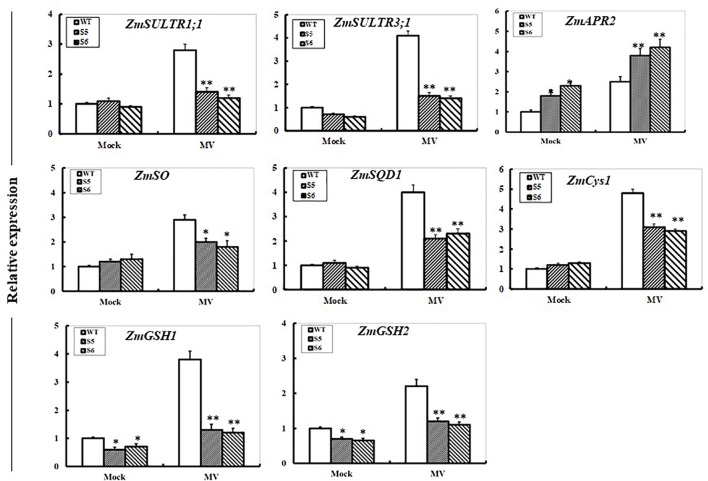
Effect of *ZmSiR* suppression on transcripts of sulfur metabolism-related genes under oxidative stress. Leaf samples from 10-day-old maize seedlings from wild-type and *ZmSiR-compromised* lines (S5 and S6) were harvested after 24 h of MV (20 μM) spraying, and then transcript levels of genes encoding sulfate transporters (*ZmSULTR1; 1* and *ZmSULTR3; 1*), ATPS reductase (*ZmAPR2*), sulfite oxidase (*ZmSO*), sulfolipid biosynthesis enzyme (*ZmSQD1*), cysteine synthetase (*ZmCys2*), and glutathione synthetase (*ZmGSH1* and *ZmGSH2*) were detected by qPCR. The mRNA levels of these genes were normalized to the transcripts of *ZmActin2* or *ZmTubulin* in the same samples. For each assay, the expression level of WT under control conditions was taken as 1.0, and data represented mean ± SE of three biological replicates. ^∗∗^*t*-test, with *P* < 0.01; ^∗^*t*-test, with *P* < 0.05.

The transcripts of S-metabolism related genes encoding sulfate transporters (*ZmSULTR1; 1* and *ZmSULTR3; 1*), APS reductase (*ZmAPR2*), sulfite oxidase (*ZmSO*), sulfolipid biosynthesis enzyme (*ZmSQD1*), cysteine synthetase (*ZmCyS2*), and glutathione synthetase (*ZmGSH1* and *ZmGSH2*) were examined under oxidative stress in WT and both RNAi maize lines by qPCR. After 24 h of MV exposure, transcripts of all the genes except for *ZmAPR2* in both RNAi plants displayed significant decreases when compared to the WT (Figure 6). In contrast, elevated expression of the *ZmAPR2* was quite clear in both RNAi maize lines compared with that in the WT. In addition, under control conditions, transcriptional expression of *ZmAPR2* was significantly up-regulated, while expression of both *ZmGSH1* and *ZmGSH2* was significantly decreased (Figure 6).

### Response of *ZmSiR-Compromised* Maize Lines Under Cold Stress

**FIGURE 7:**
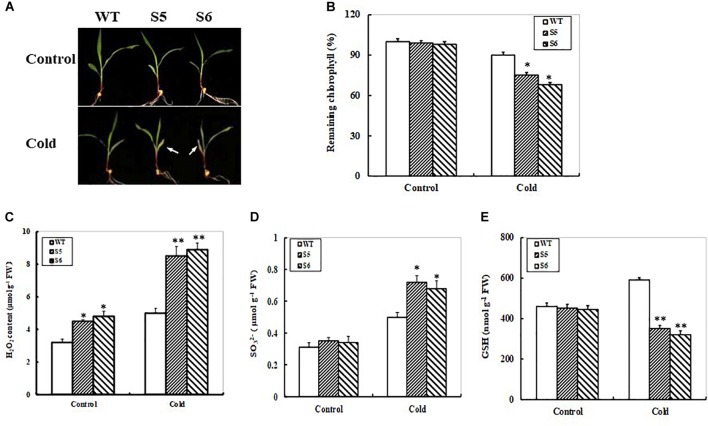
Responses of non-transgenic and *ZmSO-compromised* transgenic maize lines to cold stress. **(A)** Effect of cold stress on WT and *ZmSiR-compromised* maize seedlings. 12-day-old WT and both *ZmSiR-compromised* lines (S5 and S6) were grown hydroponically at 4°C (Cold) or 23°C (Control) for 3 days. **(B)** Relative residual chlorophyll (%) in the wild-type and *ZmSiR-compromised* lines after 3-day-cold treatment. Values are mean ± SE, *n* = 10. ^∗^*t*-test, with *P* < 0.05. **(C)** H_2_O_2_ levels were determined in the wild-type and both *ZmSiR-compromised* lines after 24 h of cold stress. Bars indicate SE (*n* = 10). Sulfite content was determined at 24 h of cold stress. **(D)** Glutathione content was measured after 24 h of cold stress. Each experiment was repeated three times. Bar indicates SE. Values are mean ± SE, *n* = 15. ^∗^*t*-test, with *P* < 0.05. **(E)** In above assays, each experiment was repeated three times. Bar indicates SE. Values are mean ± SE, *n* = 10. ^∗^*t*-test, with *P* < 0.05; ^∗∗^*t*-test, with *P* < 0.01.

Due to markedly transcriptional response of the *ZmSiR* to low temperature (Figure 1D), we tried to examine the performance of *ZmSiR-compromised* maize plants under cold stress. The hydroponic maize seedlings from WT and both *ZmSiR-compromised* lines were transferred to grow at 4°C (Cold) or 23°C (Control). After 3 days, both RNAi maize seedlings showed remarkably more leaf yellowing and necrosis than the WT (Figure 7A). In accordance with this phenotype, remaining chlorophyll in both RNAi lines was significantly lower than that in the WT (Figure 7B). This evidenced that knock-down of *ZmSiR* in maize plants also decreased cold stress tolerance.

H_2_O_2_ levels were quantitatively determined after 24-h cold stress. As shown in Figure 7C, the H_2_O_2_ levels in both *ZmSiR-compromised* lines increased by 87% averagely, while the WT only increased by 55% (Figure 7C). Furthermore, sulfite and GSH contents were determined in both RNAi and WT maize plants under cold stress. As shown in Figures 7D,E, under cold stress, sulfite levels in both RNAi maize lines increased by 100% averagely, whereas WT plants only increased by 65% (Figure 7D). Accordingly, under cold stress GSH levels in both RNAi maize lines significantly decreased, but not in the WT (Figure 7E). These results suggested that impairment of *ZmSiR* also resulted in insufficient GSH levels, and thus reduced GSH-dependent ROS scavengering capability in maize plants under cold stress. Additionally, the expression of four key S-metabolism related genes *ZmSO, ZmAPR2, ZmGSH1*, and *ZmGSH2* was examined in *ZmSiR-compromised* maize lines under cold stress by qPCR. After 24 h of cold stress, transcripts of the three genes *ZmSO, ZmGSH1*, and *ZmGSH2* displayed significant decreases, whereas *ZmAPR2* expression increased significantly in both RNAi plants when compared to the WT (Supplementary Figure S2). Moreover, transcriptional expression of *ZmAPR2* was significantly elevated even under control conditions (Supplementary Figure S2B).

## Discussion

As a key enzyme in the reductive sulfate assimilation, SiR plays important roles in sulfite detoxification, growth and development, and senescence control in plants (Khan et al., 2010; Yarmolinsky et al., 2013, 2014). In the present work, we report molecular and biochemical properties, and physiological functions of SiR from maize using several complementary approaches. Our genetic evidence suggests that ZmSiR participates in cold or oxidative stress tolerance through modulating sulfite homeostasis and GSH-mediated ROS scavenging. To our knowledge, this is the first sulfite reductase gene from monocot plants to be functionally characterized.

*SiR* exists in a single copy in *Arabidopsis* and tomato, and in two copies in poplar and rice (Bork et al., 1998; Kopriva, 2006; Brychkova et al., 2012). In maize, *ZmSiR* was found to exist only as a single gene on the 6^th^ chromosome. The established phylogenetic tree based on SiR protein sequences from 10 plant species clearly demonstrated that ZmSiR is highly similar to the known plant SiRs, suggesting *SiR* to be evolutionarily conserved in plant species. In addition to sulfite detoxification, SiR functions in growth and development, and leaf senescence regulation (Khan et al., 2010; Yarmolinsky et al., 2013, 2014). In this study, ZmSiR confers cold and oxidative stress tolerance in maize (Figures 4, 7). Together, these results indicate that plant SiR is functionally divergent.

The recombinant ZmSiR protein exhibited a sulfite-dependent sulfite reductase activity (Figure 2). The *K*_m_ of ZmSiR for sulfite was calculated to be 5.32 μM, which is much lower than the *K*_m_ values for *Arabidopsis* (8.49 μM) and tomato (19.68 μM) (Brychkova et al., 2012), suggesting that ZmSiR has higher affinity toward sulfite than SiR proteins from the two model plants (*Arabidopsis* and tomato). Further studies are needed to determine the active site responsible for substrate binding and catalytic activity in the ZmSiR protein by site-directed mutagenesis.

Methyl viologen (paraquat) can rapidly enter chloroplasts of leaves and then disrupt PSI electron transfer and produce ROS (Lewinsohn and Gressel, 1984; Babbs et al., 1989). As a key ROS molecule, hydrogen peroxide (H_2_O_2_) is induced by various abiotic stresses including MV and cold stress, and damages cellular macro-molecules such as lipids, proteins, and DNA (Bowler et al., 1994; Gechev et al., 2006). Sulfite (SO_3_^2-^) is reduced by SiR to form sulfide (S^2-^), and then the S^2-^ is converted into sulfur-containing compounds such as reduced glutathione (GSH) (Alscher, 1989; Noctor et al., 1998; Nagalakshmi and Prasa, 2001). In this study, *ZmSiR* knock-down transgenic maize plants accumulated higher H_2_O_2_ levels, but lower GSH content under cold or MV treatments (Figures 5, 7), indicating that amounts of GSH were affected by the SiR activity and in return, GSH levels largely influence H_2_O_2_ accumulation, and thus resulting in cold or oxidative stress responses of the *ZmSiR* transgenic maize plants directly. Therefore, it can speculate that ZmSiR is involved in cold and oxidative stress responses in maize possibly through modulating GSH-dependent H_2_O_2_ scavenging. In support of this viewpoint, Ding et al. (2009) have reported that transgenic tobacco plants with impaired glutathione reductase decreased MV-induced oxidative stress tolerance because of reduced glutathione regeneration ability (Ding et al., 2009). Similarly, Wang et al. (2013) have shown that down-regulation of rice SPX domain gene *OsSPX1* caused high sensitivity to cold and oxidative stresses, but the sensitivity phenotypes of rice plants could be rescued by addition of GSH, suggesting that GSH-dependent H_2_O_2_ scavenging plays an important role in cold and oxidative stress tolerance of plants (Wang et al., 2013).

Plant SiR potentially competes with two other enzymes for sulfite conversion: UDP- sulfoquinosovyl synthase (SQD1) for sulfolipid synthesis in plastids (Benning, 2007) and sulfite oxidase (SO) for sulfite oxidation in peroxisomes (Brychkova et al., 2007). As the key APR isoform, APR2 catalyzes the reduction of activated sulfate in APS to sulfite that is further reduced by SiR in leaves (Khan et al., 2010). Both SULTR1; 1 and SULTR3; 1 are two types of sulfate transporters, responsible for sulfate transport in vasculature and plastids of leaves, respectively (Cao et al., 2013; Huang et al., 2018). CyS2 and GSH1/2 are important rate-limiting enzymes for cysteine and glutathione synthesis, respectively (Droux, 2004). During cold or oxidative stress, transcripts of all these S-metabolism related genes, except for *ZmAPR2*, in *ZmSiR*-impaired maize plants displayed significant decreases when compared to the WT (Figure 6 and Supplementary Figure S2). The coordinative down-regulation might be achieved by feedback signals caused by changes in sulfur-metabolites (sulfite and GSH) in the reductive sulfate assimilation pathway. Noticeably, transcript levels of the *ZmAPR2* were significantly up-regulated in the *ZmSiR*-impaired maize lines during cold or oxidative stress compared to those in the WT plants (Figure 6 and Supplementary Figure S2). Moreover, sulfite content significantly increased in the leaves of *ZmSiR* knock-down maize lines during cold or oxidative stress (Figures 5C, 7D). These observations suggest that toxic sulfite accumulation in *ZmSiR*-impaired maize plants could be attributable to the reduced *SiR* coupled to increased *ZmAPR2* expression. In accordance with this conclusion, it was reported that constitutive expression of a bacterial *APR* in *Arabidopsis* resulted in more than threefold sulfite accumulation and showed chlorotic symptoms of toxicity, suggesting that APR over-expression induced massive de-regulation of primary sulfur metabolism (Tsakraklides et al., 2002). Together, *ZmSiR* is indispensable for maintaining sulfite homeostasis in maize during cold or oxidative stress.

In summary, ZmSiR has a sulfite-dependent SiR activity with higher affinity toward sulfite. Down-regulation of ZmSiR caused more sulfite and H_2_O_2_ accumulations, but less GSH levels, and high sensitivity to cold and oxidative stresses in maize. ZmSiR can protect plants from cold and oxidative stresses, and has a potential for engineering environmental stress-tolerant maize varieties in molecular breeding. Future work will be interesting to explore the mechanisms by which *ZmSiR* is involved in cold or oxidative stress signaling using *ZmSiR* mutant lines in maize.

## Author Contributions

ZX designed the research and wrote the manuscript. ZX, MW, and ZXu performed the research and conducted data analyses.

## Conflict of Interest Statement

The authors declare that the research was conducted in the absence of any commercial or financial relationships that could be construed as a potential conflict of interest.

## Supplementary Material

Figure S1 Genetic complementation of growth defect of the *atsir* mutant *Arabidopsis*. **(A)** Structure of the *AtSiR* locus with the T-DNA insertion site in the Salk_047219 (*atsir_s047*) mutant. The insertion site is marked by a white arrow, exons are indicated as white boxes, and untranslated regions by black boxes. P1, forward primer; P2, reverse primer; LBb1, primer specific to the T-DNA left border. **(B)** Transcript levels of *AtSiR* in the wild-type and *atsir_s047* determined by qPCR. **(C)** Relative SiR activity in leaf extracts from wild-type and *atsir_s047* plants measured by kinetic assays. **(D)** Relative activity levels of ZmSiR in the *atsir-s047* and transgenic *Arabidopsis* lines. SiR activity levels of three homozygous *atsir/35S-ZmSiR* transgenic lines (named *atsir/35S-ZmSiR* #1, #3, and #7), along with the *atsir-s047* mutant and wild-type (WT), were determined by spectrophotometry. **(E)** Representative growth phenotypes of 2-week-old seedlings from *atsir-s047* and transgenic *Arabidopsis* lines. **(F)** Dry weight of 2-week-old individual seedlings from *atsir-s047* and transgenic *Arabidopsis* lines. In the Figures **(B–F)**, asterisks on the histograms indicate significance of the difference from the corresponding control values determined by Student’s *t*-test (^∗∗^*P* < 0.01), ^∗^*t*-test, with *P* < 0.05.

Figure S2 Effect of *ZmSiR* suppression on transcript levels of several key sulfur metabolism-related genes under cold stress. Leaf samples from 12-day-old maize seedlings from wild-type and *ZmSiR-compromised* lines (S5 and S6) were harvested after 24 h of 4°C (Cold) or 23°C (Control) treatment, and transcript levels of *ZmSO* **(A)**, *ZmAPR2* **(B)**, *ZmGSH1* **(C)**, and *ZmGSH2* **(D)** were detected by qPCR as described above. Data represented mean ± SE of three biological replicates. ^∗∗^*t*-test, with *P* < 0.01; ^∗^*t*-test, with *P* < 0.05.

Table S1 PCR primers used in this study.

Table S2 Percentages of amino acid sequence identities of SiRs between maize and other plant species.

## References

[B1] Arnon D. I. (1949). Copper enzymes in isolated chloroplasts: polyphenoloxidase in *beta vulgaris*. *Plant Physiol.* 24 1–15. 10.1104/pp.24.1.1 16654194 PMC437905

[B2] Alscher R. G. (1989). Biosynthesis and antioxidant function of glutathione in plants. *Physiol. Plant.* 77 457–464. 10.1111/j.1399-3054.1989.tb05667.x

[B3] Babbs C. F. Pham J. A. Coolbaugh R. C. (1989). Lethal hydroxyl radical production in paraquat-treated plants. *Plant Physiol.* 90 1267–1270. 10.1104/pp.90.4.1267 16666920 PMC1061880

[B4] Benning C. (2007). Questions remaining in sulfolipid biosynthesis: a historical perspective. *Photosynth. Res.* 92 199–203. 10.1007/s11120-007-9144-6 17334828

[B5] Bork C. Schwenn J. D. Hell R. (1998). Isolation and characterization of a gene for assimilatory sulfite reductase from Arabidopsis thaliana. *Gene* 212 147–153. 10.1016/S0378-1119(98)00155-3 9661674

[B6] Bowler C. Van Camp W. Van Montagu M. Inzé D. (1994). Superoxide dismutase in plants. *Crit. Rev. Plant Sci.* 13 199–218. 10.1080/07352689409701914

[B7] Brychkova G. Xia Z. Yang G. Yesbergenova Z. Zhang Z. Davydov O. (2007). Sulfite oxidase protects plants against sulfur dioxide toxicity. *Plant J.* 50 696–709. 10.1111/j.1365-313X.2007.03080.x 17425719

[B8] Brychkova G. Yarmolinsky D. Ventura Y. Sagi M. (2012). A novel in-gel assay and an improved kinetic assay for determining in vitro sulfite reductase activity in plants. *Plant Cell Physiol.* 53 1507–1516. 10.1093/pcp/pcs084 22685081

[B9] Bustin S. A. Benes V. Garson J. A. Hellemans J. Huggett J. Kubista M. (2009). The MIQE guidelines: minimum information for publication of quantitative real-time PCR experiments. *Clin. Chem.* 55 611–622. 10.1373/clinchem.2008.112797 19246619

[B10] Cao M. J. Wang Z. Wirtz M. Hell R. Oliver D. J. Xiang C. B. (2013). SULTR3;1 is a chloroplast-localized sulfate transporter in *Arabidopsis thaliana*. *Plant J.* 73 607–616. 10.1111/tpj.12059 23095126

[B11] Clough S. J. Bent A. F. (1998). Floral dip: a simplified method for Agrobaterium -mediated transformation of Arabidopsis thaliana. *Plant J.* 16 735–743. 10.1046/j.1365-313x.1998.00343.x 10069079

[B12] Ding S. Lu Q. Zhang Y. Yang Z. Wen X. Zhang L. (2009). Enhanced sensitivity to oxidative stress in transgenic tobacco plants with decreased glutathione reductase activity leads to a decrease in ascorbate pool and ascorbate redox state. *Plant Mol. Biol.* 69 577–592. 10.1007/s11103-008-9440-3 19043665

[B13] Droux M. (2004). Sulfur assimilation and the role of sulfur in plant metabolism: a survey. *Photosynth. Res.* 79 331–348. 10.1023/B:PRES.0000017196.95499.11 16328799

[B14] Gechev T. S. Van Breusegem F. Stone J. M. Denev I. Laloi C. (2006). Reactive oxygen species as signals that modulate plant stress responses and programmed cell death. *Bioessays* 28 1091–1101. 10.1002/bies.20493 17041898

[B15] Huang Q. Wang M. Xia Z. (2018). The *SULTR* gene family in maize (*Zea mays L.*): gene cloning and expression analyses under sulfate starvation and abiotic stress. *J. Plant Physiol.* 220 24–33. 10.1016/j.jplph.2017.10.010 29145069

[B16] Kang Y. W. Lee J. Y. Jeon Y. Cheong G. W. Kim M. Pai H. S. (2010). In vivo effects of NbSiR silencing on chloroplast development in *Nicotiana benthamiana*. *Plant Mol. Biol.* 72 569–583. 10.1007/s11103-009-9593-8 20047069

[B17] Khan M. S. Haas F. H. Samami A. A. Gholami A. M. Bauer A. Fellenberg K. (2010). Sulfite reductase defines a newly discovered bottleneck for assimilatory sulfate reduction and is essential for growth and development in *Arabidopsis thaliana*. *Plant Cell* 22 1216–1231. 10.1105/tpc.110.074088 20424176 PMC2879758

[B18] Kobayashi Y. Otani T. Ishibashi K. Shikanai T. Nishimura Y. (2016). C-Terminal region of sulfite reductase is important to localize to chloroplast nucleoids in land plants. *Genome Biol. Evol.* 8 1459–1466. 10.1093/gbe/evw093 27189994 PMC4898807

[B19] Kopriva S. (2006). Regulation of sulfate assimilation in Arabidopsis and beyond. *Ann. Bot.* 97 479–495. 10.1093/aob/mcl006 16464881 PMC2803671

[B20] Leustek T. Martin M. N. Bick J. A. Davies J. P. (2000). Pathways and regulation of sulfur metabolism revealed through molecular and genetic studies. *Annu. Rev. Plant Physiol. Plant Mol. Biol.* 51 141–165. 10.1146/annurev.arplant.51.1.141 15012189

[B21] Leustek T. Saito K. (1999). Sulfate transport and assimilation in plants. *Plant Physiol.* 120 637–644. 10.1104/pp.120.3.637 10398698 PMC1539218

[B22] Lewandowska M. Sirko A. (2008). Recent advances in understanding plant response to sulfur-deficiency stress. *Acta Biochem. Pol.* 55 457–471. 18787711

[B23] Lewinsohn E. Gressel J. (1984). Benzyl viologen-mediated counteraction of diquat and paraquat phytotoxicities. *Plant Physiol.* 76 125–130. 10.1104/pp.76.1.125 16663782 PMC1064241

[B24] Livaka K. J. Schmittgen T. D. (2001). Analysis of relative gene expression data using real-time quantitative PCR and the 2^-ΔΔC_T_^ method. *Methods* 25 402–408. 10.1006/meth.2001.1262 11846609

[B25] Nagalakshmi N. Prasa M. N. (2001). Responses of glutathione cycle enzymes and glutathione metabolism to cooper stress in *Scenedesmus bijugatus*. *Plant Sci.* 160 291–299. 10.1016/S0168-9452(00)00392-7 11164601

[B26] Nakayama M. Akashi T. Hase T. (2000). Plant sulfite reductase: molecular structure, catalytic function and interaction with ferredoxin. *J. Inorg. Biochem.* 82 27–32. 10.1016/S0162-0134(00)00138-0 11132635

[B27] Noctor G. Arisi A. M. Jouanin L. Kunert K. J. Rennenberg H. Foyer C. H. (1998). Glutathione: biosynthesis, metabolism and relationship to stress tolerance explored in transformed plants. *J. Exp. Bot.* 49 623–647. 10.1093/jxb/49.321.623

[B28] Sekine K. Fujiwara M. Nakayama M. (2007). DNA binding and partial nucleoid localization of the chloroplast stromal enzyme ferredoxin: sulfite reductase. *FEBS J.* 274 2054–2069. 10.1111/j.1742-4658.2007.05748.x 17371503

[B29] Su X. Wei F. Huo Y. Xia Z. (2017). Comparative physiological and molecular analyses of two contrasting flue-cured tobacco genotypes under progressive drought stress. *Front. Plant Sci.* 8:827. 10.3389/fpls.2017.00827 28567053 PMC5434153

[B30] Tsakraklides G. Martin M. Chalam R. Tarczynski M. C. Schmidt A. Leustek T. (2002). Sulfate reduction is increased in transgenic *Arabidopsis thaliana* expressing 5’-adenylylsulfate reductase from *Pseudomonas aeruginosa*. *Plant J.* 32 879–889. 10.1046/j.1365-313X.2002.01477.x 12492831

[B31] Wang C. Wei Q. Zhang K. Wang L. Liu F. Zhao L. (2013). Down-regulation of OsSPX1 causes high sensitivity to cold and oxidative stresses in rice seedlings. *PLoS One* 8:e81849. 10.1371/journal.pone.0081849 24312593 PMC3849359

[B32] Wang M. Jia Y. Xu Z. Xia Z. (2016). Impairment of sulfite reductase decreases oxidative stress tolerance in *Arabidopsis thaliana*. *Front. Plant Sci.* 7:1843. 10.3389/fpls.2016.01843 27994615 PMC5133253

[B33] Xia Z. Sun K. Wang M. Wu K. Zhang H. (2012). Overexpression of a maize sulfite oxidase gene in tobacco enhances tolerance to sulfite stress via sulfite oxidation and CAT-mediated H_2_O_2_ scavenging. *PLoS One* 7:e37383. 10.1371/journal.pone.0037383 22693572 PMC3365070

[B34] Xia Z. Wei Y. Sun K. Wu J. Wang Y. Wu K. (2013). The maize AAA-type protein SKD1 confers enhanced salt and drought stress tolerance in transgenic tobacco by interacting with Lyst-interacting protein 5. *PLoS One* 8:e69787. 10.1371/journal.pone.0069787 23894539 PMC3722157

[B35] Xia Z. Wu K. Zhang H. Wu J. Wang M. (2015). Sulfite oxidase is essential for timely germination of maize seeds upon sulfite exposure. *Plant Mol. Biol. Rep.* 33 448–457. 10.1007/s11105-014-0760-y

[B36] Yarmolinsky D. Brychkova G. Fluhr R. Sagi M. (2013). Sulfite reductase protects plants against sulfite toxicity. *Plant Physiol.* 161 725–743. 10.1104/pp.112.207712 23221833 PMC3561015

[B37] Yarmolinsky D. Brychkova G. Kurmanbayeva A. Bekturova A. Ventura Y. Khozin-Goldberg I. (2014). Impairment in sulfite reductase leads to early leaf senescence in tomato plants. *Plant Physiol.* 165 1505–1520. 10.1104/pp.114.241356 24987017 PMC4119034

