# Supplementary figures and images for: The Maize Sulfite Reductase Is Involved in Cold and Oxidative Stress Responses
Source: Front Plant Sci. 2018 Nov 15;9:1680. doi: 10.3389/fpls.2018.01680 (PMC6249382; doi:10.3389/fpls.2018.01680)

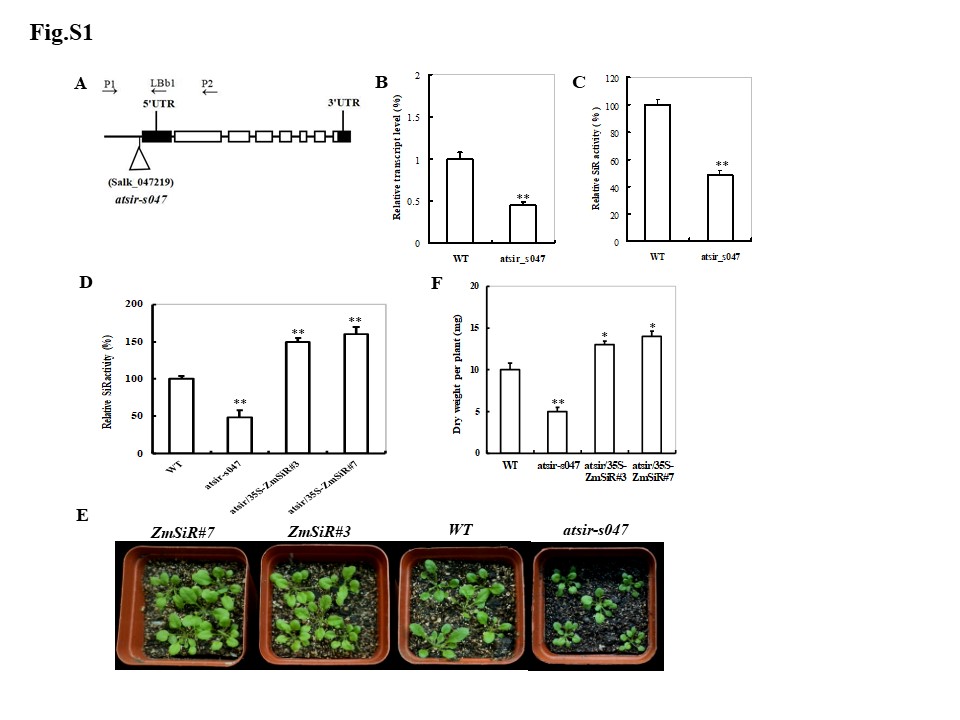

Supplement: Figure S1 — Genetic complementation of growth defect of the atsir mutant Arabidopsis. (A) Structure of the AtSiR locus with the T-DNA insertion site in the Salk_047219 (atsir_s047) mutant. The insertion site is marked by a white arrow, exons are indicated as white boxes, and untranslated regions by black boxes. P1, forward primer; P2, reverse primer; LBb1, primer specific to the T-DNA left border. (B) Transcript levels of AtSiR in the wild-type and atsir_s047 determined by qPCR. (C) Relative SiR activity in leaf extracts from wild-type and atsir_s047 plants measured by kinetic assays. (D) Relative activity levels of ZmSiR in the atsir-s047 and transgenic Arabidopsis lines. SiR activity levels of three homozygous atsir/35S-ZmSiR transgenic lines (named atsir/35S-ZmSiR #1, #3, and #7), along with the atsir-s047 mutant and wild-type (WT), were determined by spectrophotometry. (E) Representative growth phenotypes of 2-week-old seedlings from atsir-s047 and transgenic Arabidopsis lines. (F) Dry weight of 2-week-old individual seedlings from atsir-s047 and transgenic Arabidopsis lines. In the Figures (B–F), asterisks on the histograms indicate significance of the difference from the corresponding control values determined by Student’s t-test (∗∗P < 0.01), ∗t-test, with P < 0.05. [file Image_1.JPEG]

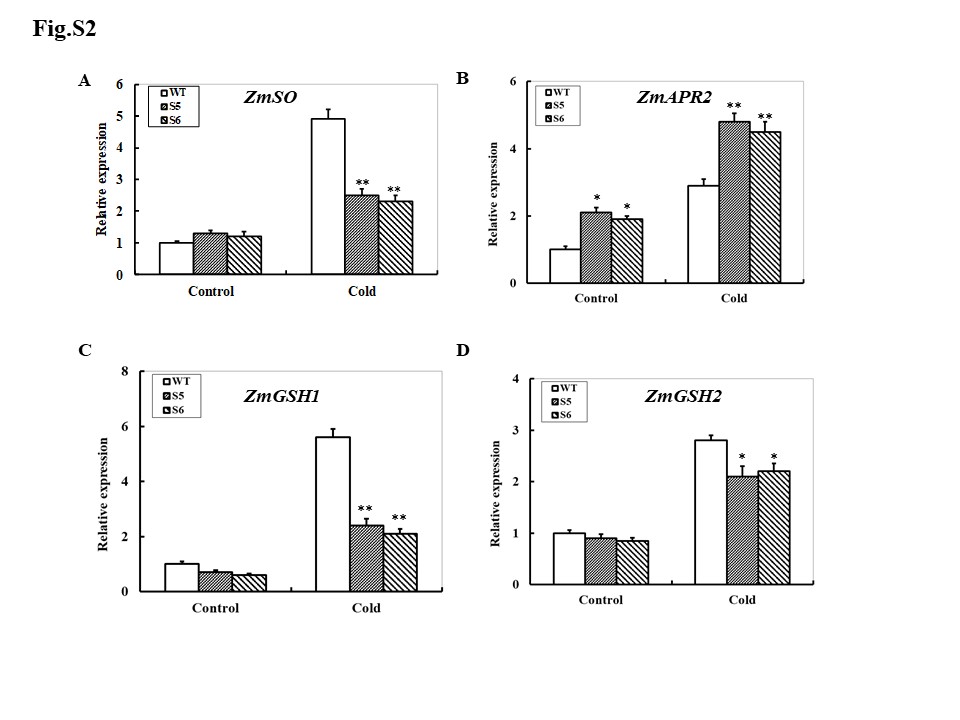

Supplement: Figure S2 — Effect of ZmSiR suppression on transcript levels of several key sulfur metabolism-related genes under cold stress. Leaf samples from 12-day-old maize seedlings from wild-type and ZmSiR-compromised lines (S5 and S6) were harvested after 24 h of 4°C (Cold) or 23°C (Control) treatment, and transcript levels of ZmSO (A), ZmAPR2 (B), ZmGSH1 (C), and ZmGSH2 (D) were detected by qPCR as described above. Data represented mean ± SE of three biological replicates. ∗∗t-test, with P < 0.01; ∗t-test, with P < 0.05. [file Image_2.JPEG]
