# Supplementary material for: The Maize Sulfite Reductase Is Involved in Cold and Oxidative Stress Responses
Source: Front Plant Sci. 2018 Nov 15;9:1680. doi: 10.3389/fpls.2018.01680 (PMC6249382; doi:10.3389/fpls.2018.01680)
Supplement: Table S1 — PCR primers used in this study. [file Data_Sheet_1.PDF]

Table S1 PCR primers used in this study

Primer name	Primer sequence*	Use
P1	5'-GTTTTGTGATAACGGCAGTGG-3'	Verification of
P2	5'-CAGTGGAGACGGCTTGAATAG-3'	homozygous atsir T-DNA
LBb1	5'-ATTTTGCCGATTTCGGAAC-3'	SALK line
ZmSiR-QF	5'-GGAAGAAGTTTGAAAGTTTCCG-3'	qPCR for *ZmSiR*
ZmSiR-QR	5'-CTTCTCAATTATCTCTCGTAGAGTTTTC-3'	expression
ZmSiR-F1	5′-ACGAATTCATGTCGGGGGCGATTG-3′	Construction of *ZmSiR*
ZmSiR-R1	5′-AGGGATCCTCATGCGGCTGACGGTGAC-3′	over-expression vector for transgenic *Arabidopsis*
ZmSiR-F2	5'-TTCTCTAGACCATGGACATTGCAGCACTTCTTACACC-3'	Construction of expression vectors for transgenic
ZmSiR-R2	5'-AAGGATCCATTTAAATAGCCCGAAACCTATCTATTCCC-3'	RNAi maize
ZmSiR-F3	5′-ACCATATGTCGGGGGCGATTG-3′	Construction of *ZmSiR*
ZmSiR-R3	5′-TACTCGAGTGCGGCTGACGGTGAC-3′	expression vector in *E.coli*
ZmSO-QF	5'-AAAGACATCAGGTCCCTCCCAAAGTA-3'	qPCR for *ZmSO*
ZmSO-QR	5'-CAATAGCAGAAACATCCCATCCAAC-3'	
ZmSULTR1.1-QF	5'- ATCAACCCACCTTCAGCTAGTCT-3'	qPCR for *ZmSULTR1.1*
ZmSULTR1.1-QR	5'-TCTTTGTTCCCATCTATCTGGTAATC-3'	expression
ZmSULTR3.1-QF	5'-GCTTCATCAGCAAGAGGCGTCCCA-3'	qPCR for *ZmSULTR3.1*
ZmSULTR3.1-QR	5'-CCGATCACTTCGATGCCGTGGTTTT-3'	expression
ZmCyS2-QF	5'-CCTCACATCGCTTTCTGACCTTT-3'	qPCR for *ZmCys2*
ZmCyS2-QR	5'-CGTTGTCCTTCTGGGCTATTCTC-3'	expression
ZmAPR2-QF	5'-GCCCGTCCGAATCCAAACCAAGT-3'	qPCR for *ZmAPR2*
ZmAPR2-QR	5'-CATCGTAGTGAGTCTGTCCCTATCCA-3'	expression
ZmGSH1-QF	5'-TTTCTTGGCCTTGGCTCCCTTGT-3'	qPCR for *ZmGSH1*

ZmGSH1-QR	5'-GCTGGAGTGCTTGTCCGTGTTGG-3'	expression
ZmGSH2-QF	5'-ACAGGAGCAACACTGCCCACGAC-3'	qPCR for *ZmGSH2*
ZmGSH2-QR	5'-TCAACAACACCAAGCCGCCAATC-3'	expression

*The underlined nucleotides constitute *Bam*HI (GGATCC), *Eco*RI (GAATTC), *Nco*I (CCATGG), *Xba*I (TCTAGA) ,*Nde* I (CATATG), *Xho*I (CTCGAG), or *Swa*I (ATTTAAAT) restriction enzyme digestion sites.
