# Supplementary material for: The Maize Sulfite Reductase Is Involved in Cold and Oxidative Stress Responses
Source: Front Plant Sci. 2018 Nov 15;9:1680. doi: 10.3389/fpls.2018.01680 (PMC6249382; doi:10.3389/fpls.2018.01680)
Supplement: Table S2 — Percentages of amino acid sequence identities of SiRs between maize and other plant species. [file Data_Sheet_2.PDF]

Table S2 Percentages of amino acid sequence identities of SiRs between maize and other plant species.

SiR	GenBank no.	Identity (%)
		Zea Mays
Zea Mays	NP_001105302.1	100
Arabidopsis thaliana	CAA89154.1	71
Solanum lycopersicum	AFB83709.1	65
Ricinus communis	XP_002513495.1	67
Nicotiana benthamiana	ACN23794.1	66
Glycine max	XP_003540209.1	68
Sorghum bicolor	XP_002441346.1	97
Oryza sativa	NP_001055978.1	91
Hordeum vulgare	BAK03240.1	89
Brachypodium distachyon	XP_003568157.1	89
